# Supplementary material for: Variant Allele of ALDH2, rs671, Associates with Attenuated Post-Vaccination Response in Anti-SARS-CoV-2 Spike Protein IgG: A Prospective Study in the Japanese General Population
Source: Vaccines (Basel). 2022 Jun 28;10(7):1035. doi: 10.3390/vaccines10071035 (PMC9320019; doi:10.3390/vaccines10071035)
Supplement: Supplementary file 1 [file vaccines-10-01035-s001.zip › vaccines-1756846-supplementary.pdf]

Supplementary Information

Table S1. Estimated correlation coefficients for fixed effects on log-transformed anti-S1 IgM, BAU/mL.

	AIC = 1341.1<br>503 observations<br>88 subjects	
Fixed effects	β	*p-value*
BNT162b2 (reference)	(reference)	
mRNA-1273	0.057	0.7132
Week 0 (reference)	(reference)	
Week 1	0.718	0.0015
Week 2	3.263	<.0001
Week 3	3.509	<.0001
Week 4	4.436	<.0001
Week 5	4.237	<.0001
Week 6	3.909	<.0001
Week 7	3.878	<.0001
Week 8	3.670	<.0001
Week 11	2.819	<.0001
Week 15	2.027	<.0001
Week 16	1.985	<.0001
Age (per year old)	-0.011	0.0201
Female sex	-0.281	0.0525
Body height (per cm)	-0.020	0.0137
Smoking status	0.202	0.2574
Ethanol intake (per category)	-0.034	0.6635
Exercise habit (per category)	-0.053	0.1866
Perceived stress (per category)	0.009	0.782
Steroid use	-0.766	0.0126
Allergic disease	0.030	0.7448
Dyslipidemia	-1.511	<.0001
ALDH2 (per variant allele)	-0.006	0.9275

β, Partial correlation coefficient

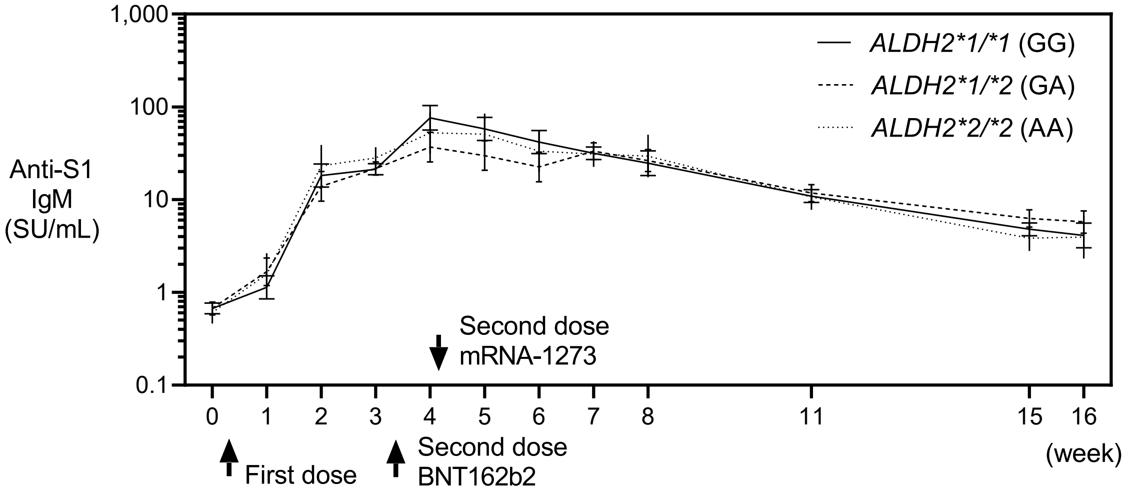

Figure S1. Estimated anti-S1 IgM antibody levels by *ALDH2* rs671 genotype.

Least-squares geometric means and standard errors were computed as shown in Figure 1.
